# A Route for Investigating Psoriasis: From the Perspective of the Pathological Mechanisms and Therapeutic Strategies of Cancer

**DOI:** 10.3390/ijms241814390

**Published:** 2023-09-21

**Authors:** Xingkang Wu, Yushuang Ma, Lu Wang, Xuemei Qin

**Affiliations:** Modern Research Center for Traditional Chinese Medicine, The Key Laboratory of Chemical Biology and Molecular Engineering of Ministry of Education, Shanxi University, No. 92, Wucheng Road, Taiyuan 030006, China; mys18834164047@163.com (Y.M.); lulu9875321@163.com (L.W.)

**Keywords:** psoriasis, cancer, cell proliferation, immune microenvironment

## Abstract

Psoriasis is an incurable skin disease that develops in about two-thirds of patients before the age of 40 and requires lifelong treatment; its pathological mechanisms have not been fully elucidated. The core pathological process of psoriasis is epidermal thickening caused by the excessive proliferation of epidermal keratinocytes, which is similar to the key feature of cancer; the malignant proliferation of cancer cells causes tumor enlargement, suggesting that there is a certain degree of commonality between psoriasis and cancer. This article reviews the pathological mechanisms that are common to psoriasis and cancer, including the interaction between cell proliferation and an abnormal immune microenvironment, metabolic reprogramming, and epigenetic reprogramming. In addition, there are common therapeutic agents and drug targets between psoriasis and cancer. Thus, psoriasis and cancer share a common pathological mechanisms–drug targets–therapeutic agents framework. On this basis, it is proposed that investigating psoriasis from a cancer perspective is beneficial to enriching the research strategies related to psoriasis.

## 1. Introduction

Psoriasis is one of the most common chronic skin diseases, with clinical features of skin lesions that have raised, well-demarcated, erythematous oval plaques with adherent silvery scales [1]. In addition to skin lesion-related symptoms, psoriatic patients suffer from comorbidities, such as psoriatic arthritis, metabolic syndrome, psychological disorders, cardiovascular disease, atherosclerosis, inflammatory bowel disease, chronic obstructive pulmonary disease, etc. [2]. Psoriasis predominantly develops in young adults, with about two-thirds of patients developing the disease before the age of 40 years, about one-third of patients developing the disease in childhood (age < 18 years), and about 57% of patients suffering from moderate to severe psoriasis [3,4]. In brief, psoriasis is a disease that seriously endangers human health.

Psoriasis is not yet curable and requires lifelong treatment. The choice of treatment for psoriasis depends on several factors, including location, the severity of the disease, and whether it is accompanied by other diseases [5]. Topical therapy involving corticosteroids, vitamin D analogues, tapinarof, and calcineurin inhibitors is the gold standard for patients with mild psoriasis [6]. Oral systemic therapies are the conventional treatment options for patients with moderate to severe psoriasis, with oral systemic agents such as methotrexate, acitretin, ciclosporin, dimethyl fumarate, apremilast, and tofacitinib [7]. Biological therapies are increasingly used to treat moderate to severe, refractory, and special types of psoriasis [8]. Currently, biologics targeting TNF-α, IL-12/IL-23 p40, IL-23p19, IL-17A, and IL-36R are used in the clinical treatment of psoriasis, and they are superior in terms of efficacy than nonbiological treatment [9,10]. Phototherapy can be safely combined with other therapeutic options to improve the effects of therapy [11]. Oral small-molecule targeted drugs are promising options for treating psoriasis, providing advantages over biologics in terms of patient convenience, reduced healthcare costs, and improved quality of life [12]. Currently, the available oral small-molecule targeted drugs for treating psoriasis include the phosphodiesterase 4 (PDE4) inhibitor apremilast and the tyrosine-protein kinase 2 (TYK2) inhibitor deucravacitinib [12]. In summary, a range of treatment methods for psoriasis has been developed over the past few decades. Despite the existence of many therapeutical options, psoriasis still cannot be completely cured owing to treatment failure and disease relapse. Therefore, there is an urgent need to investigate the molecular and genetic mechanisms of psoriasis pathogenesis from different perspectives in order to develop new therapeutic targets and drugs.

**Figure 1:**
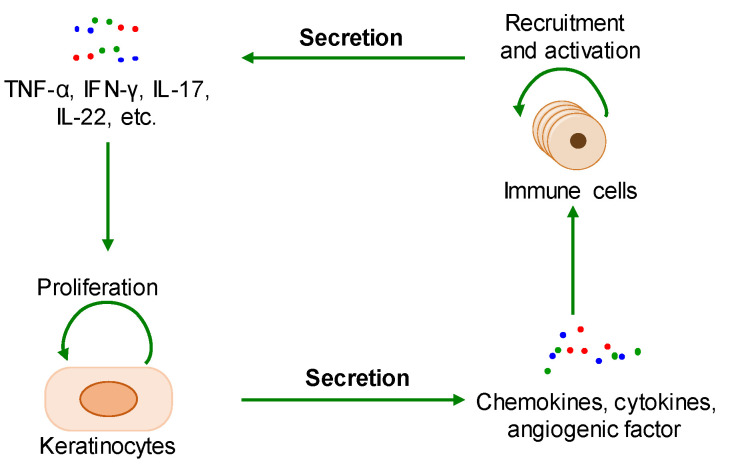
The positive feedback loop of a keratinocyte-immune microenvironment. TNF-α: tumor necrosis factor alpha; INF-γ: interferon-gamma; IL-17: interleukin-17; IL-22: interleukin-22.

The pathological mechanisms of psoriasis have not been fully elucidated. The key pathological features of psoriasis are excessive keratinocyte proliferation, abnormal differentiation, and the infiltration of a variety of immunoinflammatory cells. Additionally, its accepted pathogenesis is that the interaction between hyperproliferative epidermal keratinocytes and activated immune cells interact to form a positive feedback loop (Figure 1) [5,13]. The pathological changes related to psoriasis are similar to the key feature of cancer—that is, the malignant proliferation of cancer cells causes tumor enlargement, suggesting that there is a certain degree of commonality between psoriasis and cancer [13,14]. This review shows that psoriasis and tumors share a common pathological mechanisms–drug targets–therapeutic agents framework, leading us to propose that the research achievements of cancer provide a reliable approach for investigating the pathological mechanism and therapeutic strategies of psoriasis.

## 2. Common Pathological Mechanisms between Psoriasis and Cancer

A great deal of research has shown that the interaction between cell proliferation and abnormal immune microenvironment, metabolic reprogramming, and epigenetic reprogramming are common pathological mechanisms between psoriasis and cancer, and there are common pathogenic genes or proteins in these shared pathological mechanisms.

### 2.1. The Interaction between Cell Proliferation and Abnormal Immune Microenvironment

**Figure 2:**
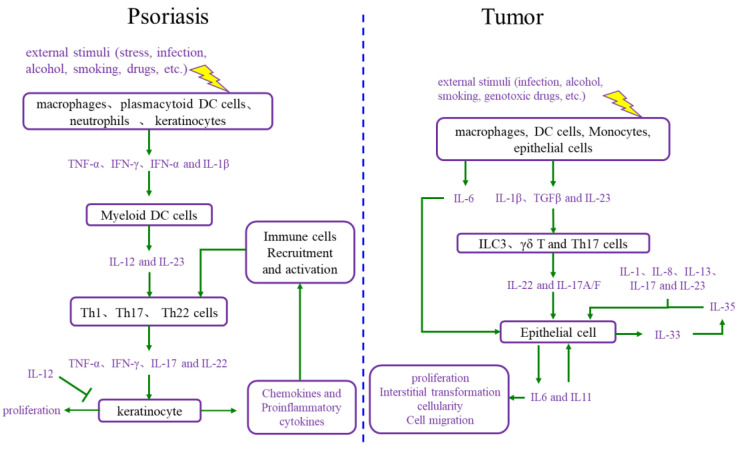
Comparison of the interaction between cell proliferation and immune microenvironment in psoriasis and cancer. DC cells: dendritic cells; IL-12: interleukin-12; IL-23: interleukin-23; Th1 cells: T helper 1 cells; Th17 cells: T helper 17 cells; Th22 cells: T helper 22 cells; TNF-α: tumor necrosis factor alpha; INF-γ: interferon gamma; IL-17: interleukin-17; IL-6: interleukin-6; IL-1β: interleukin-1β; TGFβ: transforming growth factor-β; ILC3 cells: group 3 innate lymphoid cells; γδ T cells: gamma delta T cells; IL-1: interleukin-1; IL-8: interleukin-8; IL-13: interleukin-13; IL-35: interleukin-35; IL-33: interleukin-33; IL-11: interleukin-11.

During the onset of psoriasis, external stimuli trigger the recruitment and activation of immune cells to secrete cytokines such as IL-17, IL-22, TNF-α, and INF-γ, which promote keratinocyte proliferation. The proliferative keratinocytes secrete cytokines, chemokines, and angiogenic factors such as TNF-α, IL-8, CXCL8, and VEGF, which promote angiogenesis and the recruitment and activation of immune cells. Thus, the immune microenvironment of psoriatic lesions is characterized by angiogenesis, increasing proinflammatory factors, and immune cells. Additionally, external stimuli also induce keratinocyte proliferation and the secretion of proinflammatory factors [15,16]. In summary, the proliferative keratinocytes and activated immune cells form a positive loop mediated by proinflammatory factors, maintaining and accelerating the process of psoriasis (Figure 2).

The immune microenvironment is a hallmark of cancer, and the tumor immune microenvironment is more complex than the psoriatic immune microenvironment [14]. The tumor immune microenvironment includes a set of cell types, such as endothelial cells, pericytes, immune inflammatory cells, and cancer-associated fibroblasts [17]. These cells secrete IL-6, IL-23, IL-22, IL-17, IL-1β, and TGF-β, causing cancer cell proliferation. Moreover, as the foundation of cancer, proliferative cancer cells also produce IL-6, IL-11, and other cytokines, leading to malignant proliferation and producing matrix metalloproteinases (MMPs), VEGF, and other adhesion factors, which promote angiogenesis for the nutrition supplementation of tumor cell proliferation and tumor cell circulation, as well as immune cell recruitment and activation [18,19]. Ultimately, the tumor immune microenvironment and proliferative cancer cells form a positive loop, driving tumor progression forward.

### 2.2. Metabolic Reprogramming

Metabolic reprogramming is another hallmark of cancer. The metabolic pattern of starved rapidly proliferating tumor cells changes in order to meet nutritional requirements [20]. Tumor cell proliferation mainly depends on glycolysis, referred to as the Warburg effect. To maintain the Warburg effect, the expression of glucose transporter 1 (GLUT1), pyruvate kinase (PK), hexokinase (HK), and phosphoglycerate mutase (PGAM) increases in tumors. Meanwhile, the TCA cycle is reprogrammed by the upregulation and mutation of fumarate hydratase (FH), succinate dehydrogenases (SDHs), and isocitrate dehydrogenase 1/2 (IDH1/2). In addition, the changes in enzymes such as glutamine transporter, glutaminase (GLS1), arginine succinate synthase (ASS1), phosphoglycerate dehydrogenase (PHGDH), phosphoserine aminotransferase 1 (PSAT1), serine hydroxymethyltransferase 1/2 (SHMT1/2), and indoleamine 2, 3-dioxygenase 1/2 (IDO1/2) allows for the metabolic reprogramming of amino acids such as glutamine, arginine, serine, and tryptophan metabolic pathways. Sphingomyelin metabolites are also involved in tumor development [21]. In summary, the glucose, amino acid, and lipid metabolisms are reprogrammed in cancer.

The glucose, amino acid, and lipid metabolisms also undergo metabolic reprogramming in psoriasis patients. Hyperproliferative keratinocytes are subjected to the Warburg effect. GLUT1 is required for glucose uptake by proliferative keratinocytes, and its expression is increased in hyperproliferative keratinocytes [22]. Pyruvate kinase isozyme type M2 (PKM2) is generally increased in psoriasis patients and proliferates keratinocytes to enhance glycolysis [23]. IL-17 downregulates protein phosphatase 6 (PP6) and induces the generation of arginase-1 (ARG1) in psoriasis [24]. The deficiency of PP6 leads to the accumulation of AGR1, promoting skin inflammation by driving polyamine production. The IL-17A/MALT1/c-Jun axis induces the abnormal expression of GLS1 in immune cells and keratinocytes [25]. Sphingolipid metabolite sphingosine-1-phosphate (S1P) is increased in psoriasis patients [26].

### 2.3. Epigenetic Reprogramming

Epigenetics refers to heritable molecular processes whereby a constant DNA sequence produces variable gene expression patterns without any mutational change in the DNA sequence. Compared to normal cells, tumor cells exhibit epigenetic changes such as DNA methylation, histone modifications, and genome-wide changes in the three-dimensional (3D) chromatin structure representing epigenetic mutations [27,28]. In tumor cells, alterations in the activity of epigenetic-related proteins such as histone deacetylases (HDACs), bromodomain proteins (BRDs), TET protein demethylases, histone lysine methyltransferases (HMTs), and lysine-specific demethylases (LSDs) result in abnormal histone acetylation and methylation; DNA methyltransferases (DNMT) aberrantly affect DNA/RNA methylation [27,28]. These epigenetic reprogrammings cause features such as excessive cell proliferation, death resistance, immune escape, genomic instability, and microbiome polymorphisms.

In psoriasis, epigenetic reprogramming occurs by affecting enzymes such as HADCs, BRDs, and HMTs, resulting in abnormal histone acetylation and methylation, as well as abnormal DNA methylation. It has been reported that global DNA methylation is increased in psoriasis patients, and genes with hypermethylated promoters enrich gene ontology processes, such as cell development and differentiation, actin cytoskeleton organization, cell adhesion, and motility; meanwhile, genes with hypomethylated promoters enrich immune activation processes, including inflammation, T cell activation, cytokine production, and cell proliferation [29,30]. Methylation modifications at CpG sites are increased in psoriatic lesions [31]. The promoter of the p16INK4a gene is methylated in the epidermis of psoriasis patients, and p16INK4a mRNA expression is also elevated, with high degrees of methylation of the promoter region and hypomethylation of some other regions also occurring in CD4^+^ cells [32]. Enhancer homolog 2 of the Zeste gene (EZH2) is a histone H3K27 methylase, whereas EZH2 acts on kallikrein-8 (KLK8) to cause the abnormal proliferation of keratinocytes in psoriasis [32].

### 2.4. Others

Keratin 17 (KRT17) plays an important role in psoriasis and cancer. In psoriasis, epidermal keratinocytes express KRT17, which may be recognized by DCs to trigger their own activation and maturation; mature DCs further secrete inflammatory factors and induce immature T cells to differentiate into Th1 and Th17 cells, which produce large amounts of IL-17 and IL-22 to induce epidermal keratinocyte proliferation [33,34]. KRT17 is also an oncogene that is highly expressed in cancers such as oral squamous cell carcinoma, breast cancer, and cervical cancer. It is a diagnostic marker that distinguishes malignant cervical lesions from non-malignant lesions or normal mucosa, regulates cell proliferation through interactions with 14-3-3σ, and may induce cancer cell proliferation by activating the cell growth pathway Akt/mTOR [35,36].

Long non-coding RNAs (LncRNAs) can regulate various normal immune functions and play an important role in psoriasis and tumors. In psoriasis, lncRNA PSORS1C3 is an important psoriasis susceptibility gene in patients with psoriasis vulgaris. The expression of lncRNA metastasis-associated lung adenocarcinoma transcript 1 (MALAT-1) expression is significantly increased in the lesional skin, non-lesional skin, and serum of psoriasis patients, and MALAT-1 dysregulation depends on nuclear factor kappa-B (NF-κB) [37,38]. In tumor cells, lncRNAs act via multiple mechanisms, such as chromatin remodeling, chromatin interactions, competitive endogenous RNAs (ceRNAs), and natural antisense transcripts, and lncRNA HOTAIR interacts with the cancer-associated histone complex polycomb repressive complex 2 (PRC2) to change chromatin’s structure. LncRNA HULC is highly upregulated in hepatocellular carcinoma, decreases the expression of microRNA-372, and induces the phosphorylation of the cyclic adenosine monophosphate (cAMP) response element CRE-binding protein CREB [39].

Reactive oxygen species (ROS) are major markers in the inflammatory response and affect many inflammatory pathways in various diseases. ROS drive the development of psoriasis by regulating mitogen-activated protein kinase (MAPK), NF-κB, activator protein-1 (AP-1), and the Janus kinase signal transducer and activator of transcription (JAK-STAT) signaling pathways, which are key pathogenic signaling pathways in psoriasis [40,41]. In tumor cells, ROS are also increased. Many studies have shown that NADPH oxidases (NOXs), a family of ROS enzymes, are highly expressed in tumor cells and produce ROS by responding to inflammatory mediators. ROS scavenging enzymes such as superoxide dismutase (SOD), glutathione peroxidase, and peroxidase change; moreover, tumor suppressor gene mutations such as p53 mutation cause an increase in ROS in tumors [42,43].

## 3. Common Therapeutic Agents and Therapeutic Targets in Psoriasis and Cancer

### 3.1. Current Therapeutic Agents and Their Targets

At present, many drugs have shown therapeutic effects in psoriasis and tumors by targeting the same target (Table 1).

#### 3.1.1. Cytotoxic Agents Regulating the Cell Cycle

The inhibitors of human topoisomerase I and II are conventional cytotoxic agents for the treatment of cancer and psoriasis [44,45]. Human DNA topoisomerase I is essential for DNA replication and is expressed at much higher levels in proliferative cells. Camptothecin, a topoisomerase I inhibitor, is a first-line anticancer drug that has been developed as a topical drug for psoriasis treatment since the early 1970s in China [46,47]. Human DNA topoisomerase II (Topo II) comprises two subtypes: Topo IIα and Topo IIβ. Topo IIα is a key protein required for cell proliferation and Topo IIβ is not related to cell proliferation [48]. Two antitumor Topo II inhibitors, bimolane and ICRF-154, were used in the oral treatment of psoriasis from 1973 to 2006 in China [49,50]. The approved Topo II inhibitors cannot selectively inhibit Topo IIα, but they can inhibit Topo IIβ, causing DNA damage in normal cells [51,52]. Small molecule inhibitors targeting cyclin-dependent kinases (CDKs) play an important role in the evolving field of anticancer treatment [53]. CDK7 expression was markedly increased in CD4^+^ T cells from patients with psoriasis, and its inhibitor THZ1 ameliorated psoriasiform symptoms in an imiquimod-induced psoriasis-like mouse model [54]. Paclitaxel is a chemotherapeutic agent targeting microtubules, and micellar paclitaxel has demonstrated therapeutic activity in patients with severe psoriasis [55,56]. Methotrexate is an antitumor drug that mainly inhibits DNA synthesis by inhibiting dihydrofolate reductase, thereby inhibiting the growth of tumor cells [57]. Methotrexate is also a commonly used therapeutic agent in psoriasis; its anti-psoriasis mechanism is complex and includes inhibitory effects on folate-dependent enzyme, NF-κB, lincRNA-p21, NO synthase, and solute-carrier family 46 member 2 (SLC46A2) [58].

#### 3.1.2. Immune Modulators

The JAK/STAT signaling pathway plays a critical role in the signaling of a wide array of cytokines and growth factors, and the dysregulation of the JAK/STAT pathway is associated with various cancers and autoimmune diseases [59]. Currently, at least nine small-molecule inhibitors of the JAK/STAT signaling pathway are in development for moderate to severe psoriasis and psoriatic arthritis [60,61]. Deucravacitinib is a first-in-class TYK2 small molecule inhibitor that received approval in the USA on 9 September 2022 for the oral treatment of adults with moderate to severe plaque psoriasis [62]. The pharmacologic inhibition of TYK2 with deucravacitinib decreased proliferation and induced apoptosis over time in malignant peripheral nerve sheath tumors in a dose-dependent manner [63]. The biological targeted agent tildrakizumab specifically antagonizes IL-23 and targets the p19 subunit of IL-23 to inhibit inflammation; it has been used in the clinical treatment of psoriasis while regulating the tumor microenvironment and has been in phase I/II clinical studies for the treatment of prostate cancer [64,65]. The CJM112 monoclonal antibody targets IL-17 to inhibit inflammation and is in phase I clinical studies for the treatment of psoriasis, multiple myeloma, colon cancer, and breast cancer [66,67,68].

#### 3.1.3. Epigenetic Modulators

JQ1, which targets BRD4 and exhibits therapeutic utility in cancer, inhibited the proliferation of keratinocytes and modulated the RORC/IL-17A pathway in a mouse model of psoriasis-like inflammation [69,70,71,72]. The p300/CBP inhibitor A485 displays selective inhibitory activity against several hematological malignancies and androgen receptor-positive prostate cancer [73]. A485 normalizes psoriatic fibroblast gene expression in vitro and reduces psoriasis-like skin inflammation in vivo [74]. Vorinostat, an anti-tumor epigenetic modulator commonly used in the treatment of hematologic cancer, targets HDACs to inhibit keratinocyte proliferation and is undergoing preclinical studies for the treatment of psoriasis [75,76]. Decitabine, a DNA methyltransferase inhibitor, is used to treat myelodysplastic syndromes and ameliorates the imiquimod-induced mouse model of psoriasis [77,78].

#### 3.1.4. Metabolic Modulators

GLUT1 is identified as a novel therapeutic target for psoriasis, and its topical inhibition with WZB117 decreases psoriasiform hyperplasia by inhibiting keratinocyte proliferation in mouse models of psoriasis-like disease [22]. GLUT1 is also a promising drug target for cancer treatment [79]. WZB117 has been shown to significantly inhibit A549 tumor growth over a period of 10 weeks [80]. PKM2, a valuable therapeutic target for cancer, mediates IL-17 signaling in keratinocytes driving psoriatic skin inflammation [81,82]. 2′-Hydroxycinnamaldehyde (HCA), the active component isolated from the stem bark of *Cinnamomum cassia*, exerts anticancer effects through multiple mechanisms and ameliorates imiquimod-induced psoriasiform inflammation by targeting PKM2-STAT3 signaling in mice [83,84].

#### 3.1.5. Others

VEGF antagonists target VEGF to inhibit angiogenesis and have been used in the clinical treatment of tumors. They have shown clinical efficacy in the treatment of psoriasis, as evidenced by a case report that VEGF antagonists lead to a significant improvement in the psoriasis of metastatic cancer patients [85,86]. Calcipotriol, a vitamin D receptor agonist, is the topical gold standard for the treatment of psoriasis and is able to reverse cisplatin resistance and enhance the efficacy of PD1 antibodies in the treatment of pancreatic ductal adenocarcinoma in gastric cancer [87]. Hsp90 is a highly abundant and ubiquitous molecular chaperone that plays an essential role in cell proliferation; it is a hot topic in the research and development of anti-tumor drugs. Hsp90 inhibitor RGRN-305 has been found to relieve psoriatic lesions in cancer patients in phase I clinical trials for the treatment of cancer, and it has shown good efficacy and safety in phase Ib clinical trials for the treatment of plaque psoriasis [88,89,90]. T-LAK cell-oriented protein kinase (TOPK) is a marker of tumor progression because it is a potent promoter of the malignant proliferation of tumor cells. The TOPK inhibitor OTS514 induces the cytokinesis defect of cancer cells, suggesting that the inhibition of TOPK may be a viable therapeutic option for the treatment of various cancers [91]. TOPK levels are predominantly upregulated in the epidermal keratinocytes of psoriatic lesions in both psoriasis patients and model mice and are positively associated with psoriasis progression. TOPK was upregulated in psoriatic lesions. The topical application of OTS514 clearly alleviates epidermal hyperplasia by inducing G_2_/M phase arrest and the apoptosis of keratinocytes [92]. All of this indicates that more and more studies have shown that some drugs exhibit effectiveness in the treatment of psoriasis and tumors.

### 3.2. Novel Potential Therapeutic Targets

More and more studies have shown that some proteins are becoming novel potential therapeutic targets for psoriasis and tumors. KRT17 is overexpressed in both psoriatic keratinocytes and tumor cells, and the topical application of KRT17 siRNA relieves psoriasiform lesions in mice and exhibits antitumor effects in gastric cancer cells [93,94]. TWEAK is a cytokine, and TWEAK and its receptor Fn14 are upregulated in cancer and promote cancer progression. The TWEAK antibody RG7212 has been in clinical trials for the treatment of cancer [95,96,97]. TWEAK is elevated in the serum and skin lesions of psoriasis patients, and the TWEAK antibody and knockout of Fn14 have shown an alleviating effect on psoriasis in mice [98]. Proprotein convertase subtilisin-kexin type 9 (PCSK9) is a sensitive gene in psoriasis, and its protein is elevated in the skin lesions and serum of psoriasis patients; knockout of PCSK9 slows the development of psoriasis-like skin lesions and inflammation in mice [99,100,101]. PCSK9 expression is elevated in a variety of cancers, and knockout of PCSK9 inhibits tumor cell proliferation, induces apoptosis, inhibits tumor growth in mice, and prolongs survival in tumor-bearing mice [102]. Angiopoietin-like protein 4 (ANGPTL4) is highly expressed in the skin lesions of psoriasis patients, ANGPTL4 recombinant protein promotes psoriasis-like skin lesions in mice, and the knockout of ANGPTL4 inhibits keratinocyte growth and expresses cytokines such as IL-17, IL-6, and TNF-α [103]. ANGPTL4 is highly expressed in pancreatic cancer tumor tissues and is associated with the progression of pancreatic cancer. The overexpression of ANGPTL4 promotes pancreatic cancer tumor growth in mice, and ANGPTL4 knockdown inhibits pancreatic cancer tumor growth in mice [104]. The above studies indicate that KRT17, TWEAK, PCSK9, and ANGPTL4 have the potential to serve as therapeutic targets for psoriasis and tumors and require the development of nucleic acid drugs, biologics, and small-molecule drugs against them.

**Table 1:** Summary of common therapeutic agents in psoriasis and cancer.

Drugs		Targets	Clinical Phase		Ref
			Psoriasis	Cancer	
Cytotoxic agents	Camptothecin	Topoisomerase I	Clinical application (topical)	Clinical application for various cancers	[46,47]
	Bimolane and ICRF-154	Topoisomerase II	Clinical application (oral)	Clinical application for various cancers	[49,50,51,52]
	THZ1	CDK7	Preclinical study (topical)	Preclinical study	[53,54]
	Paclitaxel	Microtubule	Clinical application (topical)	Clinical application for various cancers	[55,56]
	Methotrexate	Folate-dependent enzyme	Clinical application	Clinical application for various cancers	[57,58]
Immune modulators	Deucravacitinib	TYK2	Clinical application (oral)	Preclinical study for MPNST	[62,63]
	Tildrakizumab	IL-23	Clinical application	Clinical phase I/II for prostatic cancer	[64,65]
	CJM112	IL-17	Clinical phase I	Clinical phase I for colorectal cancer	[66,67,68]
Epigenetic modulators	JQ1	BRD4	Preclinical study	Clinical phase I for melanoma	[69,70,71,72]
	A485	p300/CBP	Preclinical study	Preclinical study for hematological malignancies and prostate cancer	[73,74]
	Vorinostat	HDACs	Preclinical study	Clinical application for hematologic cancer	[75,76]
	Decitabine	DNMTs	Preclinical study	Clinical application for MDS	[77,78]
Metabolic modulators	WZB117	GLUT1	Preclinical study	Preclinical study for lung cancer	[22,79,80]
	2′-Hydroxycinnamaldehyde	PKM2	Preclinical study	Preclinical study for various cancers	[83,84]
Others	VEGF antagonist	VEGF	Proven effective based on clinical observation	Clinical application for various cancers	[85,86]
	Calcipotriol	VDR	Clinical application (topical)	Preclinical study for gastric cancer	[87]
	RGRN-305	Hsp90	Clinical phase Ib	Clinical phase I for various cancers	[88,89,90]
	OTS514	TOPK	Preclinical study (topical)	Preclinical study for various cancers	[91,92]

Annotation: CDK7: cyclin-dependent kinase 7; TYK2: tyrosine protein kinase 2; IL-23: interleukin-23; IL-17: interleukin-17; BRD4: bromo domain-containing protein 4; p300/CBP: histone acetyltransferase paralogues/ CREB-binding protein; HDACs: histone deacetylases; DNMTs: DNA methyltransferases; GLUT1: glucose transporter 1; PKM2: pyruvate kinase isozyme type M2; VEGF: vascular endothelial growth factor; VDR: vitamin D receptor; Hsp90: heat shock protein 90; TOPK: T-LAK cell-oriented protein kinase; MPNST: malignant peripheral nerve sheath tumors; MDS: myelodysplastic syndromes.

## 4. Discussion

As an incurable skin disease, psoriasis constitutes a significant burden for patients, having a huge impact not only on patients’ skin and organs but also on their spirit. Although the number of studies related to psoriasis has gradually increased since around 2003, the research content is not comprehensive enough, and the pathological mechanisms of the disease have not been elucidated clearly or in sufficient depth. The treatment of psoriasis is also a significant problem; no drug can comprehensively cure psoriasis and many drugs can only reduce and control symptoms, meaning that long-term treatment is necessary. The psoriasis population tends to be younger, which generates higher requirements for patient compliance, as well as the efficacy and safety of psoriasis treatments. However, even the most effective biologic agents carry the risk of infection during use, and the tolerability and safety of long-term use have not been established. Moreover, prolonged psoriasis can also cause cardiovascular disease, metabolic syndrome, mental illnesses, and other complications; the long-term harm it causes cannot be ignored. Therefore, in recent years, doctors and patients have come to no longer simply regard psoriasis as a skin problem, and more and more attention has been paid to the study of psoriasis. This article reviewed the latest pathological mechanism and drug discoveries of psoriasis and found that psoriasis and tumors have common pathological mechanisms, therapeutic drugs, and therapeutic targets. We propose studying the pathological mechanisms and therapeutic strategies of psoriasis from an oncological perspective.

The core pathological process of psoriasis is epidermal thickening caused by the excessive proliferation of epidermal keratinocytes, and this pathological change is similar to the key feature of tumors—the malignant proliferation of tumor cells, causing tumor enlargement. Abnormal cell proliferation interacting with the immune microenvironment, angiogenesis, metabolic reprogramming, epigenetic reprogramming, the abnormal expression of the KRT17 protein, non-coding RNA, and elevated ROS are all common pathological mechanisms of psoriasis and tumors, and there are common causative genes or proteins in these shared pathological mechanisms. At the same time, there are many common therapeutic agents for psoriasis and tumors, and the efficacy of these drugs is usually achieved through the same target protein—for example, calcipotriol, the topical gold standard drug for psoriasis, activates vitamin D receptor-sensitizing chemotherapeutic drugs and immunotherapy in some tumors, and topical HDAC inhibitors, which are antineoplastic drugs, have the effect of treating psoriasis. Furthermore, several proteins, such as KRT17, KRT17, TWEAK, PCSK9, and ANGPTL4, are highly expressed in psoriasis and tumors and are involved in the development of these two diseases, and knockdown of these proteins exhibits therapeutic effects. Therefore, there are common pathological mechanisms, therapeutic agents, and therapeutic targets in psoriasis and tumors, and it is feasible to study psoriasis from an oncological perspective.

Because malignant tumors are fatal diseases, the research results for tumor pathogenesis and treatment strategies are among the most numerous, with approximately 70 times more papers than for psoriasis in PubMed. With the help of omics techniques such as genomics, proteomics, transcriptomics, protein epigenetics, and metabolomics, as well as the study of molecular cell biology, a variety of molecular mechanisms that regulate the biological characteristics of tumors (including gene mutations, cell signal transduction, metabolic reprogramming, epigenetic reprogramming, etc.) have been revealed and elucidated. These mechanisms have a guiding role in dissecting the omics analysis results of psoriasis and are conducive to the study of the pathological mechanism and drug targets of psoriasis. At the same time, there are many kinds of marketed therapeutic drugs for cancer, and there are countless candidate drugs and potential drugs that provide a basis, in terms of drugs, for formulating personalized therapeutic strategies based on different tumor types and patients; they also hold reference significance for solving the problem of large differences in individual efficacy in the treatment of psoriasis. Therefore, based on the remarkable accumulation of oncology research, studying the pathogenesis of psoriasis and its therapeutic drugs from the perspective of oncology is conducive to using oncology research to improve the efficiency of psoriasis research.

However, as two complex chronic diseases, psoriasis and tumors also have their own distinct characteristics. Resisting cell death is one of the most significant hallmarks of cancer cells [105]. The physiological apoptosis of keratinocytes maintains the homeostasis of the skin, and psoriatic keratinocytes exhibit decreased apoptosis [106]. Ferroptosis is a programmed cell death induced by ferrous iron overload and the accumulation of lipid peroxidation. Ferroptosis activation of keratinocytes is involved in the progression of psoriatic lesions; ferroptosis inhibitor ferrostatin-1 significantly abated the phenotype of IMQ-induced psoriatic lesions in mice [107,108]. Inducing ferroptosis has developed as a novel potential avenue for cancer treatment, as evidenced by several cell lines and unique cancer cell states exhibiting intrinsic sensitivity to ferroptosis [109]. GSDMD-mediated pyroptosis is activated in keratinocytes of psoriatic lesion; both GSDMD knockout and pyroptosis inhibitor disulfiram alleviate imiquimod-induced psoriasis-like lesions in mice [110]. Inducing nonapoptotic cells to pyroptosis is considered a potential cancer treatment strategy to overcome chemotherapy resistance [111]. Galectin-7 expression is low in psoriasis and enhances keratinocyte sensitivity to IL-17A [112].. High expression of levels galectin-7 promote its metastasis in T-cell lymphoma and play a role in sensitizing chemotherapy in urothelial cell carcinoma [113,114].

Particularly, not only psoriasis and tumors but almost all diseases are complicatedly associated with inflammation. Some immune cells and factors play different roles in psoriasis and cancer. Th17 cells are one subset of pro-inflammatory CD4^+^ helper T cells that orchestrate the inflammatory response to various immune stimuli [115]. Th17 cells are activated by IL-23, promoting the progression of psoriasis via IL-17 [116]. Drugs targeting IL-23/Th17 cells have successfully achieved fine curative effects for psoriasis [116]. In contrast, the role of Th17 cells in cancer pathogenesis and therapy is still controversial. Th17 cells and IL-17A have been found in various tumors. IL-17 function is either tumor promoting or tumor protective based on the type of tumor and disease stage [117,118]. TNF-α plays an important role in the pathogenesis of psoriasis, and therefore has become the primary target of modern therapy. However, its role in tumors is complex and has the effect of promoting or inhibiting tumors of different types or different stages [119]. IL-12 is an important antitumor cytokine used to enhance tumor immune response [120]. However, the understanding of the role of IL-12 in psoriasis is complex and varied. IL-12 expression is elevated in psoriatic lesions. The activation of IL-12 signaling in keratinocytes alleviates psoriasiform skin inflammation, whereas IL-12 activates Th1 cells to produce INF-γ and TNF-α, which act as surrogates for psoriasis by driving skin inflammation [121,122,123,124,125]. Therefore, IL-12 has a pivotal role in maintaining skin epithelial cell homeostasis. The activation of the aryl hydrocarbon receptor (AhR) promotes the expression of anti-inflammatory cells and anti-inflammatory factors and inhibits the immune response, and AhR inhibitors have become key therapeutic agents for the treatment of psoriasis [126]. However, AhR is overactivated in tumors, causing immune escape, and inhibiting the activity of AhR can activate the immune response; it is therefore a target in cancer immunotherapy [127]. A multicenter study found that approximately 57% of cancer patients with a history of psoriasis experienced relapsed or worsened psoriasis when treated with immune checkpoint inhibitors [128]. The above studies show that the differences between psoriasis and tumors have a significant impact on their therapeutic effects. In particular, the diversity and complexity of tumor types determine the diversification of pathological mechanisms and treatment strategies, and the question of how to undertake the study of commonalities with psoriasis from the vast research on tumor biology requires in-depth thinking and exploration.

The understanding of the interaction between cell proliferation and the abnormal immune microenvironment in psoriasis and tumors has undergone different development histories. Over the past two decades, immune cells and factors have been considered the main driver of psoriasis, whereas keratinocytes are the executors of immune function [129]. In recent years, more studies have advanced our understanding of the pathogenesis of psoriasis in terms of keratinocytes participating in both the initiation and maintenance phases of psoriasis by responding to environmental stimuli such as trauma and stress [130]. However, over the past few decades, increased cell division has been regarded as a cause of cancer via driving neoplastic transformation induced by chronic irritation, including hormones, chemicals, physical trauma, etc. [131,132]. Owing to the demonstration of two key concepts—immunosurveillance and immunoediting—the recognition of tumor immunology sprang up at the beginning of the 21st century and reached it climax at the Nobel Prize in Physiology or Medicine 2018 [133]. Meanwhile, many of the therapeutic strategies of cancer are designed to inhibit and kill proliferative cancer cells. Nevertheless, the understanding of psoriasis has undergone a process from an abnormal immune microenvironment to abnormal cell cycle dynamics. Targeting the abnormal immune microenvironment has been the mainstream strategy for the treatment of psoriasis, yet there are no approved drugs targeting the cell dynamics of keratinocytes [16,134]. Here, again, we anticipate that the signaling circuitry describing psoriatic keratinocytes will be charted in far greater detail and clarity by means of knowledge about cancer cell cycle dynamics, promoting the discovery of anti-psoriatic drugs targeting keratinocytes.

## 5. Conclusions

In summary, there is a certain degree of commonality between psoriasis and cancer. The core pathological feature of psoriasis and cancer is excessive cell proliferation driven by the abnormal immune microenvironment, metabolic reprogramming, epigenetic reprogramming, and other abnormalities (KRT17 overexpression, lncRNAs, and ROS). Meanwhile, there are common therapeutic agents between psoriasis and cancer, including cytotoxic agents regulating the cell cycle, immune modulators, epigenetic modulators, metabolic modulators, and others (VEGF antagonists, Hsp90 inhibitor RGRN-305, and calcipotriol). Moreover, novel targets without targeted agents also show promising therapeutic effects in both psoriasis and cancer. Based on these conclusions, psoriasis and cancer have a common pathological mechanisms–therapeutic targets–therapeutic agents framework.

## 6. Future Directions

Research on the pathological mechanisms of psoriasis remains insufficient, and psoriasis therapeutics are far from meeting the needs of patients. Due to the considerable accumulation of studies in oncology, the common pathological mechanisms–therapeutic targets–therapeutic agents framework between psoriasis and cancer provides a new approach for researching psoriasis. From the perspective of oncology research, researching the pathological mechanisms of psoriasis and its therapeutic drugs is conducive to enriching psoriasis research strategies.

As tumor biology is more fully understood, cellular pathways and molecules (including RNAs, proteins, metabolites, and others endogenous or exogenous molecules) that contribute to tumor development, cancer recurrence, and survival are being defined. Psoriasis is an incurable skin disease that seriously affects the physical and mental health of patients. However, the pathological mechanisms of psoriasis are far from being sufficiently understood, and there are many unclarified mechanisms. Tumor biology can provide guidance for the study of the pathological mechanism of psoriasis via the use of omics technology, systems biology, and AI technology.

It is well known that anti-tumor agents, including approved drugs, drugs in the trial phase, clinically failed drugs, candidate drugs, and lead drugs, account for a large proportion of total drugs. Based on the action mechanisms of these antitumor agents, the use of AI technology to screen anti-psoriasis agents will become an effective method for psoriasis drug research. In particular, some agents with good clinical anti-cancer effects that were withdrawn from clinical practice due to side effects can be developed as topical drugs for the treatment of psoriasis. However, the differences and similarities of psoriasis and tumors need to be deeply researched and elucidated. One idea that has guiding significance for the study of anti-tumor drugs relates to new uses for old drugs.

## Data Availability

Not applicable.
